# An open-set hybrid CNN-TCN network for human action recognition with unknown action rejection in collaborative robot workspaces

**DOI:** 10.3389/frobt.2026.1919411

**Published:** 2026-09-11

**Authors:** Yasir Abdullah R, Ignisha Rajathi G, Barakkath Nisha U, Johny Elton R

**Affiliations:** 1 Department of AI&DS, Dr. Mahalingam College of Engineering and Technology, Pollachi, Tamil Nadu, India; 2 Manipal Institute of Technology Bengaluru, Manipal Academy of Higher Education, Manipal, India; 3 Department of CSE, Dr. Mahalingam College of Engineering and Technology, Pollachi, Tamil Nadu, India; 4 Department of CSE, Sri Krishna College of Engineering and Technology, Coimbatore, India

**Keywords:** collaborative robots, human action recognition, hybrid CNN, open set recognition, temporal modeling, unknown action rejection

## Abstract

**Introduction:**

Open-set recognition capability is becoming necessary for collaborative robots because actual shop-floor dynamics do not always correspond to the fixed action classes used during training. Misclassification of unknown human motions may lead to inappropriate robot responses, whereas reliable rejection enables safer supervisory control.

**Methods:**

An open-set human action recognition framework is developed by combining a spatial Convolutional Neural Network encoder with a Temporal Convolutional Network-based temporal aggregation module and an unknown-action rejection gate based on open-set scoring. The framework is evaluated on the NTU RGB+D 120 dataset using cross-subject and cross-view protocols with held-out action classes.

**Results:**

The proposed framework achieves 93.20% and 91.00% top-1 accuracy under cross-subject and cross-view evaluation, respectively, with corresponding Macro-F1 scores of 88.40% and 86.20%. For open-set detection, it achieves an AUROC of 0.938 and an AUPR of 0.750, with a 5.00% false acceptance rate for unknown actions and a 91.82% true known-action acceptance rate. The system operates at 15.0 ms per window and 66 fps on an NVIDIA Jetson Orin NX 16 GB, with a model size of 42 MB.

**Discussion:**

The results demonstrate that calibrated unknown-action rejection can improve safety-aware human action recognition while retaining practical real-time performance for collaborative robot workspaces.

## 1 Introduction

The capability to predict human actions and the ability to detect unexpected events in which an action performed by the human differs from the known set of behaviours are required in terms of safety. Current collaborative safety-aware assembly work has already made this link between the task context and the risk-aware robot motion explicit, noting that the lack of certainty in the perception of the action can result in unsafe motions (Yi et al., 2024). Moreover, the existing literature on human–robot multimodal interaction in smart manufacturing clearly states that the “understanding” phase should hold in scenarios affected by occlusions, viewpoint changes, and different intentions from the human (Wang T. et al., 2024). The key design features of collaborative robots include assistive nature, adaptation capabilities, and intrinsic safety, all of which are achieved through robust perception-control interactions that work reliably in spite of the real variability of operating conditions. The standards of collaborative operation stipulate requirements related to speed and separation monitoring, power and force limiting, and protective stopping, yet the criteria triggering these modes become more and more dependent upon perception output rather than tactile and proximity sensing alone (Rosenstrauch et al., 2018). If a cobot (Collaborative Robot) can understand that the person is taking a tool out of the fixture, turning the part, or entering a dangerous area, it will be able to use safe motion primitives and interrupt trajectories before proximity criteria are reached. A considerable amount of research on human-robot collaboration considers recognition of actions as an integral part of the collaborative system, since such integration allows to time the actions better, avoid unnecessary stops, and increase confidence in operation (Yu et al., 2024). However, this research indicates that recognition errors can lead to brittle behavior and frustrate operators, especially when there are many objects in the workspace. However, most approaches for video and skeleton-based HAR (Human Action Recognition) are based on a closed-set problem formulation where the test actions are assumed to share the label set of the training data. Recent benchmark studies in 2024 demonstrate that human activity/action recognition models remain affected by dataset-related bias arising from collection protocols, user demographics, sensor/device differences, and cross-dataset distribution shifts (Wang Tianyu et al., 2024), (Alarfaj et al., 2024) and (Romeo et al., 2024).

Workers perform their tasks by improvisation, rearrangement of actions, collaboration, unusual usage of tools, and sometimes unpredictable actions due to fatigue or urgency (Yin et al., 2024). The recognition algorithm which is forced to choose among the known classes could easily classify an unknown action incorrectly, and this issue is especially hazardous when control policies translate the prediction into motion modification or speed change (Dai et al., 2024). The problem is also aggravated by hardware limitations of robot controllers and IPC systems: while accurate, heavy models could be slow or unstable after quantization. Current lightweight models that leverage knowledge distillation in order to design skeleton action recognition architectures explicitly highlight the trade-off between recognition performance and deployability, highlighting the influence of real-time constraints on architecture design (Noor et al., 2024). Moreover, important testing strategies for robotic applications, such as cross-subject and cross-view generalisation, highlight weaknesses which are usually masked out by random splits; the NTU RGB + D 120 database standardised such evaluation paradigms because viewpoint and subject variation significantly impact action recognition (Liu J. et al., 2020).

### 1.1 Open-set HAR definition and relevance to robot risk-aware autonomy

Open-set HAR expands the problem formulation in the sense that the implemented model will be faced with actions which are not seen in the training labels and will hence have to (i) categorize familiar actions accurately and (ii) reject unfamiliar actions. This is especially important in robotics where the unknown label trigger safe fall-back actions including reduced velocity, increased distances, active sensing or even human verification rather than a confident label. Open-set HAR builds on the methods of OOD detection and open-set recognition, yet action recognition poses some additional challenges due to temporal windows that may comprise transitions, partial actions, multiple persons and context-dependant intent. Recent critical analysis of OOD detection and open-set recognition methods shows that performance can be strongly affected by benchmark design, scale, and distribution-shift conditions (Wang Hongjun et al., 2024). In terms of open-set recognition, modern approaches oppose treating the unknown space as one category, but rather treat unknown space more structurally to reduce the impact on distribution and improve boundary learning (Wang Hongjun et al., 2024). With regards to action recognition, the need for this is supported by open-set skeleton-based benchmark datasets that demonstrate conventional open set methods do not work well with pose sequences alone, and that additional modality information can make significant improvement in rejecting unknowns (Li et al., 2024) (Peng et al., 2024). Similar issues arise in an egocentric scenario, where open set action recognition must deal with motion blurring and sudden camera movements despite occlusions.

There are still some issues in applying them to a cobot control scenario instead of a cloud inference pipeline. Firstly, most of the open set formulations assume static image recognition tasks, while using similar rejection techniques for video window classification implies that such a model should be stable enough to avoid the oscillation of “known” and “unknown” status between subsequent frames. Secondly, safety critical policies require confidence estimation, but often calibration procedures and the choice of thresholds are performed separately from the development of the main part of the architecture. Lastly, interpretable decision making has operational value–when a robot slows down or stops because of an “unknown action”, people working in the same workspace should receive a short, auditable justification to disable their safety measures without getting annoyed. The recent developments of open-set recognition by vision-language models prove that scale cannot resolve the issue automatically (Miller et al., 2025). Fourthly, the use of robots always introduces novelty in the form of new hardware tools, new fixtures, new uniforms, and camera mounts configured in new ways. The concepts proposed in open world recognition and open world detection surveys suggest that any model should be prepared for changing label sets and support incrementally learning, but they are not applied to cobot-specific HAR pipelines (Mullappilly et al., 2024). In addition, there is room for integrating deep temporal modeling with lightweight geometry-based or hand-crafted priors, which are inherently robust to background cluttering (Nan et al., 2024). Such approaches could increase robustness of models when limited computation resources are available and make explanations easier for understanding why certain motions lead to specific predictions.

The proposed research work builds a hybrid CNN temporal network for cobot workcells that emphasizes temporal consistency, cross-view performance, and safety-aware recognition capabilities. The approach incorporates an unknown-action rejection system and a calibrated decision-making strategy to allow the robot to be cautious when observing something beyond the learned set of actions. The proposed work introduces the metrics tailored to robotics applications, which include recognition accuracy metrics as well as latency, feasibility, and safety measures. The validation of the algorithm is performed using cross-subject and cross-view experiments. The review of related literature, problem formulation, model description, datasets and metrics, ablation and failure analysis, and deployment consequences will follow. The novel aspect of this research is not the creation of CNN, TCN, energy-based scoring, or open-set recognition since these methods have already been invented. Instead, what this research does is create a risk-aware open-set human action recognition method to use for collaborative robots in which actions that are unknown or unsafe must be rejected and not forced to fit into one of the existing known classes.

This proposed method combines the aspects of spatial feature extraction, temporal action modeling, calibrated unknownness scoring, and confidence-aware risk scoring within a decision framework that can be used for safe human-robot cooperation. This method solves the problem faced by the closed-set HAR system that assumes that all actions are from the known classes. The main Contributions are as follows.

- A hybrid model that consists of both CNN and TCN is proposed for learning spatial and temporal features from human activities in collaborative workspaces.
- An energy-based unknownness estimation technique is included in order to perform open set recognition that rejects the presence of unknown/unsafe actions and does not classify them under known actions.
- A risk-based decision score is proposed by combining unknownness with decision confidence that enables more cautious decisions of actions in critical human-robotic collaboration.
- The above techniques are validated using closed-set recognition accuracy, AUROC/AUPR for unknown detection, OSCR, ablation study, and parameter sensitivity analysis.

## 2 Related work

In collaborative robotics, human action recognition (HAR) has moved beyond the problem of offline classification of actions from video data to an online perception module with safety implications that is part of closed-loop control system. When humans and robots work in a common space, an HAR component will not just be evaluated on its recognition abilities but also on its ability to manage uncertainties, partial observability and unusual behavior of humans which might provoke dangerous robot movements. Hence, the focus of recent robotics research has been on multimodal pipelines, hybrid systems which have temporal coherence and reliability metrics that can be used by autonomy layers.

### 2.1 Human action recognition for robotics and cobot perception pipelines

Robotics HAR systems are hardly used independently for classification. In collaborative work cells, recognition results are usually combined with other perception features (e.g., human pose, workspace occupancy, tool status) and further utilized by high-level decision-making modules controlling robot timing and speed scaling or collision prevention. Recent studies on human-robot collaboration in an industrial setting suggest that most HAR failures are due to occlusion, viewpoint mismatch, or temporal misalignment, rather than inadequate spatial representation only, making the need for temporally consistent HAR and proper evaluation methodology evident (Mehak et al., 2024). The dataset-based approach, in turn, has evolved toward action labeling that is task-oriented, so that each class corresponds to a certain assembly step and robot actions can be directly derived from HAR results (Büsch et al., 2025; Romeo et al., 2024; Pilacinski et al., 2024).

Another related trend is the incorporation of HAR within proactive HRC systems. Human intent inference surveys and predictive HRC surveys point out the importance of the HAR problem in being able to recognize the next step early enough so that the robot plans can be changed in advance to avoid any unsafe proximity (Hoffman et al., 2024). The same holds true for proactive human robot interaction approaches where recognition has to be considered as belief tracking under uncertainty where the lack of evidence means that the robot has to be conservative in its actions and cannot force a classification (Marike Koch van Den broek and Moeslund, 2024).

### 2.2 Hybrid CNN temporal networks

The CNN-architecture with a temporal component is still prevalent in robotics HAR due to the need for feature richness while being able to predict an inference cost. CNN architectures efficiently encode spatial features based on RGB/depth video streams and recurrence plots, whereas temporal components are essential for robot timing and gating of actions. The multimodal collaborative assembling process has shown that the combination of the information in the representation space and further application of the temporal attention or transformer block could enhance viewpoint-invariance (Feichtenhofer et al., 2020; Kondratyuk et al., 2021; Qin et al., 2025; Wang Tianyu et al., 2024). Temporal Convolutional Networks (TCNs) are appealing for resource-constrained cobots because TCNs have convolutional receptive fields which allow long-term temporal reasoning without recurring state updates, thus providing stable latency guarantees and easy parallelism (He et al., 2022; Wei and Wang, 2024). While attention models are added to TCN/GRU/LSTM blocks to highlight the discriminative phase of events (such as the hand-to-tool touch), while filtering out all other motions in the scene, attention model weights do not serve as calibrated confidence and need to be explicitly treated as such in safety-critical use cases (Shi and Liu, 2024). Mixture-of-experts and gate routing approaches have also been studied for compute allocation in temporal segments, offering interesting opportunities for edge deployment but raising another issue of reliability, namely, stability under novel and noisy situations (Wang Jing et al., 2025).

### 2.3 Open-set recognition and unknown rejection methods

Open-set recognition reinterprets HAR as a decision problem under partial information, in which the classifier must make two decisions at once, namely, what action is taking place and if the action observed is part of any known classes. One of the most important lessons learned from studies of open-set recognition and out-of-distribution detection is that the performance of rejection relies highly on the way “knownness” scores are computed and thresholds are set for different levels of openness (Guerin et al., 2023). It is a popular technique to use softmax score thresholding because it is simple yet unreliable under distribution shift. Scores derived based on energy functions, distances to prototypes, and tail models based on EVT/OpenMax techniques are widely used techniques due to their independence from the simplex normalization property of the softmax outputs (Guerin et al., 2023) (Yang et al., 2024). Techniques based on EVT try to estimate the open space risk based on statistics of extremities in the distances between features. Distance and density-based approaches consider unknown classes as improbable samples in the learned embedding space. Nonetheless, most existing OSR(Open-Set Recognition) benchmarks are not robot-specific; they hardly address multi-perspective occlusions, real-time operation, or safety-critical decision making where the rejection score implies concrete actions such as slowing down or stopping.

In the case of cobots, unknown rejection is only as good as its calibration. The rejection threshold has to remain constant irrespective of any viewpoint, operator, or variations in lighting else the robot will continue swinging between being overconfident and unsafe on one side and being prone to generating false alarms on the other. According to surveys on reliability and uncertainty in perception for safety-critical systems, calibration metrics like Expected Calibration Error (ECE), Negative Log-Likelihood (NLL) and selective risk curves ought to be considered first class citizens alongside accuracy (Araújo et al., 2024). The system-level safety analysis also stresses the importance of traceability, monitoring, and observability of the perception modules in the autonomy stack to ensure assurance and auditing after any incident (Kochanthara et al., 2024). This is especially true for open-set HAR where an “unknown” decision must be recorded along with relevant information (e.g., score, status of sensors, timing) so that the operators can check whether the robot has acted conservatively for valid reasons. The HAR using sensor data also emphasizes the actual cases where miscalibration occurs due to placement heterogeneity of sensors and noise in signals (Alarfaj et al., 2024). While hybrid temporal architectures and task-aligned datasets have been developed in recent research on HAR for robots, open set recognition is mostly performed via a threshold on closed set scores (Wei and Wang, 2024). At the same time, while the OSR/OOD community offers advanced approaches for measuring knownness, robotics benchmarks lack considerations of such robotics-specific restrictions as cross-view occlusion, latency issues, action anticipation, and auditability (Liu et al., 2020b; Kochanthara et al., 2024). Calibration has become a widely accepted element in the development of safety-critical perception, but it is still not widely reported in robotics HAR benchmarking, and there is no link between safety policies and failure detection. Additionally, recent research work has also confirmed the use of convolutional neural networks in autonomous robotics especially when used with reinforcement learning for robotic decision making based on perception (Padhiary et al., 2025). There has also been evidence of neural network-based approaches demonstrating impressive performance in high-resolution computer vision problems (Ravi Kiran et al., 2025; Zou et al., 2024). A summary of robotics-oriented HAR and open-set reliability research is presented in Table 1.

**TABLE 1:** Robotics-oriented HAR and open-set reliability research.

Research axis	Typical robotics setting	Model family trend	Unknown handling trend	Reliability focus and metrics	Identified key gaps	Representative studies
HRC dataset realism	Manufacturing assembly, workstation tasks	CNN + temporal heads	Mostly closed-set	Cross-subject/cross-view protocols	Few explicit unknown splits	(Mehak et al., 2024), (Büsch et al., 2025), (Romeo et al., 2024)
Pipeline integration	Online HRC assistance and monitoring	Perception-to-policy loops	Implicit rejection via logic	System dependability discussions	Few calibrated reject scores	(Mohammadi Amin et al., 2020), (Hoffman et al., 2024), (Marike Koch van Den broek and Moeslund, 2024)
Hybrid temporal design	Resource-bounded cobot perception	CNN–GRU/LSTM/TCN	Usually, thresholded logits	Latency, stability ablations	Uncertainty rarely safety-linked	(Wang et al., 2024c), (Wei and Wang, 2024), (Wang et al., 2025a)
Temporal attention emphasis	Early recognition, anticipation	TCN + attention/transformer	Closed-set; OOD suggested	Temporal consistency metrics	Not evaluated as open-set	(Wei and Wang, 2024), (Shi and Liu, 2024)
Open-set scoring	Generic OSR/OOD benchmarks	Feature scoring methods	Energy, distance, EVT/OpenMax	AUROC, OSCR	Robotics coupling missing	(Guerin et al., 2023), (Yang et al., 2024)
Calibration and assurance	Safety-critical perception	Monitoring + calibration	Threshold policy-driven	ECE, NLL, Brier, risk curves	Audit/logging rarely discussed	(Kochanthara et al., 2024), (Araújo et al., 2024)
Sensor-HAR for robots	Wearables and human-centric robotics	CNN + attention	Closed-set, limited OSR	Robustness under noise	Unknowns underexplored	(Alarfaj et al., 2024), (Carlos et al., 2024)

## 3 Problem formulation and design requirements

When robots perform together in workcells, action recognition be formulated as a decision-making problem under uncertainty. It is not enough for the robot to merely recognize the objects; it needs to make a decision about the extent to which it should act on the recognized action. In cases where there is insufficient evidence for the recognition, the more cautious approach is to declare the lack of certainty.

### 3.1 Notation and task definition

Let $X=\left\{X_{t}\right\}\frac{T}{t=1}$ deno te a video window of T frames sampled from a continuous stream using stride S . A spatial encoder $g\left(\cdot \right)$ maps each frame $x_{t}$ ​ to a feature vector $z_{t}=g\left(x_{t}\right)$ , producing $Z=\left\{Z_{t}\right\}\frac{T}{t=1}$ . A temporal module $h\left(\cdot \right)$ aggregates Z into a sequence descriptor $s=h\left(Z\right)$ . A classifier head $c\left(\cdot \right)$ outputs a logit vector $a\left(X\right)=\left[a_{1}\left(X\right),\ldots ,a_{K}\left(X\right)\right]$ over K known action classes, where $Y=\left\{1,\ldots ,K\right\}$ is the known label set. The model produces a predicted known label $\hat{y}\left(X\right)=\arg\max_{k\in Y}a_{k}\left(X\right)$ and an associated confidence $\hat{p}\left(X\right)=\max_{k\in Y}p\left(k|X\right)$ ​, where $p\left(\cdot |X\right)$ is obtained from $a\left(X\right)$ via a chosen normalisation or calibration step. The open-set output space extends Y with an explicit unknown label u . In addition to $\hat{y}\left(X\right)$ , the system computes a scalar “unknownness” score $n\left(X\right)$ that is designed to increase when X deviates from the known-action manifold.

### 3.2 Closed-set vs. open-set inference setting for cobots

In the closed-set setting, every window is assumed to belong to Y , and the decision is forced: $d_{\text{closed}}\left(X\right)=\hat{y}\left(X\right)$ . This assumption is brittle for cobots because operational streams include unseen behaviours, mixed or transitional motions, tool-induced occlusions, and camera viewpoint shifts. Under these conditions, a closed-set model can still return a confident known label even when the observation does not truly match any known action, which is hazardous if downstream logic maps the label directly to speed scaling, trajectory changes, or task synchronization. In the open-set setting, windows may originate from Y or from an unknown distribution U that represents actions and motion patterns outside the trained vocabulary. The decision rule must therefore allow rejection as a first-class output: $d_{\text{open}}\left(X\right)\in Y\cup \left\{u\right\}$ .

### 3.3 Open-set decision objective

The objective combines (i) accurate classification of known actions and (ii) dependable rejection of unknowns. The earlier phrase “ $n\left(X\right)$ and a threshold τ ” becomes correct only when the directionality is explicit. Here $n\left(X\right)$ is defined as an unknownness score, so larger values mean “more likely unknown.” The decision rule as follows in Equation 1

$$d\left(X\right)=\left\{\begin{array}{cc} \hat{y}\left(X\right), & \text{if }n\left(X\right)\leq \tau \\ u, & \text{if }n\left(X\right)>\tau \end{array}\right.$$ (1)

This formulation is intentionally conservative: a window is accepted as a known class only when its unknownness remains within an acceptance region. In practice, τ is not an arbitrary constant; it is selected using a validation procedure that targets a desired operating point, such as bounding the false acceptance rate of unknowns while maintaining adequate acceptance of known actions. For cobots, the operating point is often risk-asymmetric, meaning the threshold is tuned to penalise false acceptances more heavily than false rejections, especially for action classes whose misinterpretation can induce unsafe robot motion.

### 3.4 Robotics constraints

Real-time operation calls for bounded latency per window as well as constant throughput to ensure decisions are temporally aligned with robot control cycles. Hardware limitations enforce restrictions on model size, memory requirements, and buffers, which restricts the temporal context size and encourages efficient spatial backbone and temporal networks. In addition, decisions have to be temporally stable–without any smoothing or hysteresis, slight changes in n(X) around τ will result in a fast switch from known label to u, causing undesirable stop-and-go motion that reduces efficiency and user confidence. Finally, the algorithm has to be fail-safe–when the input is ambiguous or noisy, it is better to choose u than to assign a wrong label and provide the decision value n(X) for further safety rules implementation.

## 4 Proposed methodology

This subsection describes an end-to-end pipeline for open set perception that is particularly suited for collaborative robots operating in a shared workspace. The architecture combines an efficient learning process for spatiotemporal features with a known-reject policy and safety interface, where perception uncertainties can be translated into safe robot policies. The goal of such an approach is to achieve three main objectives: reliable action recognition, reliable rejection of novel actions, and bounded latency.

### 4.1 System overview

The pipeline starts with continuous perception and generates either a recognized action label or an unrecognized decision which initiates an expected safety response. A video window of fixed length is segmented out of the stream, is preprocessed to handle variations in lighting and motion, goes through a CNN-temporal network, is scored to detect open-set “unknownness,” and finally is turned into a command for the robot controller.

**FIGURE 1:**
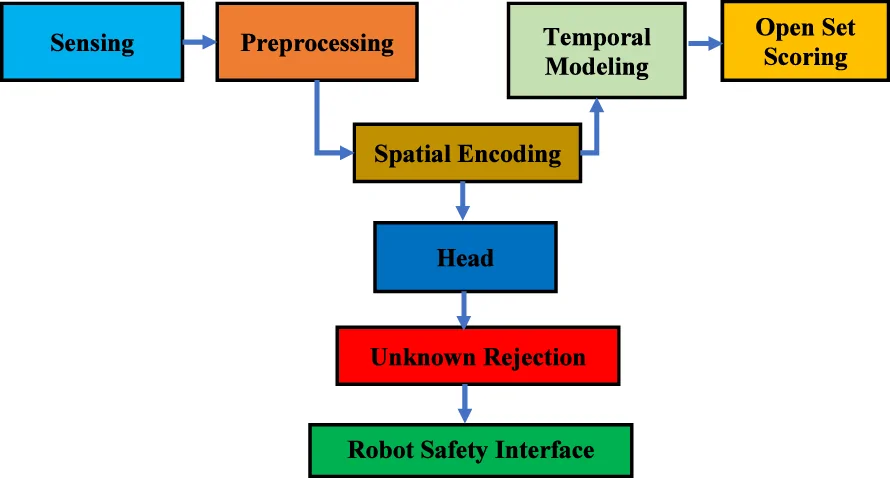
End-to-end perception-to-safety pipeline of the proposed approach, showing the flow from sensing and preprocessing through spatial encoding, temporal modeling, open-set scoring, unknown rejection, and the robot safety interface.

The perception node generates at runtime (a) the accepted action label, (b) an unknownness measure, (c) a refined confidence value, and (d) a safety status. The latter outputs serve as inputs to a safety manager that imposes hysteresis and dwell time constraints on the process to prevent stop–go cycles in the vicinity of a decision threshold. Figure 1 demonstrates the full process chain from perception to safety in order to control the collaborative robot in a shared workspace. It starts with the sensory step, in which continuous visual data are collected and forwarded to preprocessing, where the data stream is stabilized by normalization, denoising, and window creation. Spatial encoding creates compact embeddings per frame with an increased focus on human movement cues and minimized background effects. Temporal modeling merges the frame-level descriptors within the window in order to model the action dynamics and transitions necessary for separating similar workcell actions. The head translates the temporal descriptor to class probabilities which together with the open-set score are evaluated in order to measure the resemblance of the current window to the learned action space. If the open-set score suggests novelty or uncertainty, the unknown rejection step is triggered in order to avoid any forced misclassification.

### 4.2 Spatial feature encoder

The spatial encoder derives compact motion-informative descriptors from each image frame, yet stays compatible with embedded computer architecture. Two backbones are considered. A light-weight backbone is employed if a mobile CNN is used (for instance, depth-wise separable convolutions), in case of a workcell where lower latency is needed. A standard backbone is considered if the perception pipeline is executed on a discrete GPU or edge accelerator. As a means to increase awareness of motion-related information despite viewpoint changes and partial occlusion, the encoder may feature multi-scale dilated blocks. Such blocks combine different receptive fields without down sampling, thus preserving detailed hand-object interaction information. Frame-level features are produced as

$$\begin{array}{cccc} & z_{t}=g\left(x_{t};\theta_{g}\right),t=1,2,\ldots ,T & & \end{array}$$ (2)

In Equation 2, $x_{t}$ denotes the t ^th^ frame of the current window, $g\left(\cdot \right)$ is the CNN encoder, $\theta_{g}$ ​ is the encoder parameter set, T is the window length in frames, and $z_{t}\in R^{d}$ is the extracted feature vector of dimension d . To stabilize training and reduce sensitivity to illumination and camera gain, features are normalized and regularized. Normalization is applied at the feature-map level (batch or group normalization depending on batch size stability), and regularization combines spatial dropout with mild weight decay to discourage shortcut reliance on background textures that do not transfer across workcells.

### 4.3 Temporal modeling module

The temporal module constructs a window representation from the frame-level sequence, such that it is invariant to changes in speed, incomplete actions, and short-term occlusions. For the case of cobots, the Temporal Convolution Network (TCN) is chosen as the main temporal module because it has three properties: (i) bounded latency, (ii) parallelism in time steps, and (iii) controlled receptive field through dilation. In the case when the perception-to-control latency constraints are tight, this property becomes important. Temporal aggregation is represented by

$$s=h\left(\left\{Z_{t}\right\}\frac{T}{t=1};\theta_{h}\right)$$ (3)

In Equation 3, $\left\{z_{t}\right\}$ is the feature sequence from Equation 2, $h\left(\cdot \right)$ is the TCN aggregator, $\theta_{h}$ denotes temporal-module parameters, and $s\in R^{m}$ is the window descriptor of dimension m . Windowing and sampling are fixed for reproducibility. A practical configuration uses $T\in \left[16,32\right]$ frames per window with stride $S\in \left[4,8\right]$ frames to balance responsiveness and temporal context. Frame sampling uses uniform sub-sampling inside the window, with occasional speed jitter during training to reduce sensitivity to operator pace.

### 4.4 Open-set unknown rejection module

The open-set component is designed as a first-class decision layer rather than a *post hoc* threshold over closed-set softmax outputs. The module includes a known-class head, a dedicated open-set scoring function, threshold selection with calibration, and a risk-aware policy that maps uncertainty to robot actions.

#### 4.4.1 Known-class classifier head

The classifier head maps the temporal descriptor to K known classes. Logits are computed as

$$a_{k}=w_{k}^{⊤}s+b_{k},k=1,2,\ldots ,K$$ (4)

In Equation 4, s is the temporal descriptor from Equation 3, $w_{k}$ ​ and $b_{k}$ ​ are the weight vector and bias for class k , and $a_{k}$ ​ is the unnormalized logit for that class. The vector $a=\left[a_{1},\ldots ,a_{K}\right]$ drives both known-class prediction and open-set scoring. $w_{k}$ ​ and $b_{k}$ represent the weight vector and the bias parameter of the last classification layer for class k. Both b and k are optimized along with the CNN-TCN model through backpropagation. Weights are initialized through the Xavier/Glorot technique while biases are initialized to zero.

#### 4.4.2 Open-set scoring function

An energy-based score is used because it is simple, differentiable, and effective at separating in-distribution samples from unknowns without requiring an explicit unknown class. The energy score is

$$E\left(X\right)=-\tau_{T}\log\left(\sum_{k=1}^{K}\exp\left(\frac{a_{k}}{\tau_{T}}\right)\right)$$ (5)

In Equation 5, X is the current video window, $a_{k}$ ​ are logits from Equation 4, and $\tau_{T}>0$ is a temperature parameter that controls score sharpness. More negative energy typically corresponds to higher confidence in known classes, while higher energy indicates likely unknown or ambiguous windows.

To align with the “unknownness” convention used in the decision rule, the pipeline defines

$$\tilde{n}\left(X\right)=\sigma \left(\alpha E\left(X\right)+\beta \right)$$ (6)

where α>0 and β are affine calibration parameters.

In Equation 6, $E\left(X\right)$ comes from Equation 5, and $\tilde{n}\left(X\right)$ is the scalar unknownness score used for rejection; α scales the sensitivity of unknownness to energy and β shifts the operating range for consistent thresholding. For reproducibility, consider two input sequences with energy scores $E\left(X_{1}\right)=-2.50$ and $E\left(X_{2}\right)=-0.80$ . Using α=1.0 and β=0.0 , the calibrated unknownness score is computed as:

$$\tilde{n}\left(X\right)=\sigma \left(\alpha E\left(X\right)+\beta \right)$$

$$\tilde{n}\left(X_{1}\right)=\sigma \left(-2.50\right)=0.076$$

$$\tilde{n}\left(X_{2}\right)=\sigma \left(-0.80\right)=0.310$$

Thus, $X_{1}$ receives a lower calibrated unknownness score because it has stronger known-class evidence, whereas $X_{2}$ receives a higher unknownness score and is more likely to be rejected as an unknown action.

#### 4.4.3 Threshold selection and calibration

Thresholding is treated as an operating-point selection problem, not a fixed constant. A validation set is constructed with (i) known-class windows and (ii) proxy unknown windows, created either by holding out a subset of classes during threshold fitting or by applying outlier exposure transformations that break known-action structure (temporal shuffling, aggressive speed jitter, or spatial masking around hands/tools). Calibration uses temperature scaling on logits and an affine mapping on the energy-derived unknownness to improve threshold portability across viewpoints. A global threshold is used when the workcell risk is uniform, while class-conditional thresholds are enabled when certain action confusions are more hazardous.

Threshold tuning was done using a calibration data set which included 50% held-out windows labeled as belonging to certain classes and 50% outlier exposure transformed windows. The held-out windows were kept separate from the training split and thus were not used for optimization of the parameters of the model. Unknown classes used for testing were not part of the training at all. Hence, no leakage was present between training and test classes.

#### 4.4.4 Risk-aware rejection policy for cobots

The safety supervisor consumes the open-set decision and converts it into a robot state recommendation. A simple but effective policy combines unknownness with known-class confidence:

$$r\left(X\right)=\lambda \tilde{n}\left(X\right)+\left(1-\lambda \right)\left(1-\hat{p}\left(X\right)\right)$$ (7)

In Equation 7, $\tilde{n}\left(X\right)$ is from Equation 6, $\hat{p}\left(X\right)=\max_{k}p\left(k|X\right)$ is the top known-class confidence, $\lambda \in \left[0,1\right]$ controls the blend, and $r\left(X\right)$ is a risk indicator used to gate safety actions. Higher $r\left(X\right)$ implies higher uncertainty or novelty. The supervisor then applies conservative logic with hysteresis: if the system outputs unknown or the risk exceeds a high threshold for H consecutive windows, it triggers a slowdown or protective stop. If risk drops below a lower threshold for H windows, normal operation resumes. This prevents rapid toggling when the observation hovers near the boundary. Here, λ is a dimensionless risk-balancing weight that controls the contribution of the calibrated unknownness score $\tilde{n}\left(X\right)$ relative to the classification confidence. The risk score $r\left(X\right)$ represents the final open-set rejection score used to decide whether an input sequence should be accepted as a known action or rejected as unknown. A higher value of $r\left(X\right)$ indicates greater rejection risk. The quantitative effect of $r\left(X\right)$ is reflected in the AUROC, AUPR, and OSCR results reported in Section 6.2.

### 4.5 Training strategy

Training is performed in two stages: supervised learning on known actions, followed by rejection-oriented tuning using proxy unknown exposure.

#### 4.5.1 Supervised training for known classes

Known-class learning uses standard cross-entropy:

$$L_{CE}^{w}=-\frac{1}{N}\sum_{i=1}^{N}w_{y_{i}}\log\left(p_{i,y_{i}}\right)$$ (8)

$$w_{c}=\frac{N}{C\times N_{c}}$$

where N is the total number of training samples, C is the number of known training classes, $N_{c}$ is the number of samples in class c , $y_{i}$ is the ground-truth label of sample i , $p_{i,y_{i}}$ is the predicted probability for the correct class, and $w_{c}$ is the inverse-frequency weight assigned to class c . This gives larger weights to underrepresented classes and lower weights to highly frequent classes

#### 4.5.2 Synthetic unknown generation or outlier exposure

Proxy unknowns are generated without adding new semantic labels. Windows are corrupted using transformations that preserve low-level appearance but violate action structure, such as temporal segment permutation, temporal reversal, aggressive motion blur, or spatial masking focused on hands and tools. These samples teach the scorer to raise unknownness when action evidence is structurally inconsistent.

#### 4.5.3 Loss functions

An auxiliary separation loss pushes proxy unknown energy upward while keeping known energy lower:

$$\begin{array}{cccc} & L_{\text{os}}=E_{X∼D_{K}}\left[\max\left(0,E\left(X\right)-m_{K}\right)\right]+E_{X∼D_{U}}\left[\max\left(0,m_{U}-E\left(X\right)\right)\right] & & \end{array}$$ (9)

The mathematical formulation of the spatial encoder, temporal module, classifier, open-set scoring, risk-aware decision policy, and training objective is summarized in Equations 2–9. In Equation 9, $E\left(X\right)$ is defined in (5), $D_{K}$ ​ is the known training distribution, $D_{U}$ ​ is the proxy-unknown distribution, and $m_{K}<m_{U}$ ​ are margins that define desired energy ranges for known *versus* unknown samples. The total loss is $L=L_{\text{ce}}+\gamma \;L_{\text{os}}$ ​, where γ controls how strongly open-set separation influences training. The algorithm for Open-set inference and safety gating for cobot is expressed in Table 2.

**TABLE 2:** Algorithm: Open-set inference and safety gating for cobot.

Step	Operation	Output
1	Acquire frames and form window $X=\left\{x_{t}\right\}\frac{T}{t=1}$ using stride S	Window X
2	Pre process frames (resize, normalization, optional denoise)	Cleaned window
3	Compute spatial features $z_{t}=g\left(x_{t};\theta_{g}\right)$ as in (2)	Sequence Z
4	Aggregate temporally $s=h)Z;\theta_{h}$ as in (3)	Descriptor s
5	Compute logits $a_{k}=w_{k}^{⊤}s+b_{k}$ as in (4)	Logits a
6	Compute energy $E\left(X\right)$ using (5) and unknownness $n\left(X\right)$ using (6)	$E\left(X\right),n\left(X\right)$
7	Apply open-set decision rule from Section 3 using $n\left(X\right)$ and threshold τ	Label in Y or u
8	Compute risk $r\left(X\right)$ using (7) and apply hysteresis logic	Safety state
9	Publish $\hat{y}$ or u , $\hat{p}$ , $n\left(X\right)$ , $r\left(X\right)$ , and safety recommendation	Robot interface signals

**FIGURE 2:**
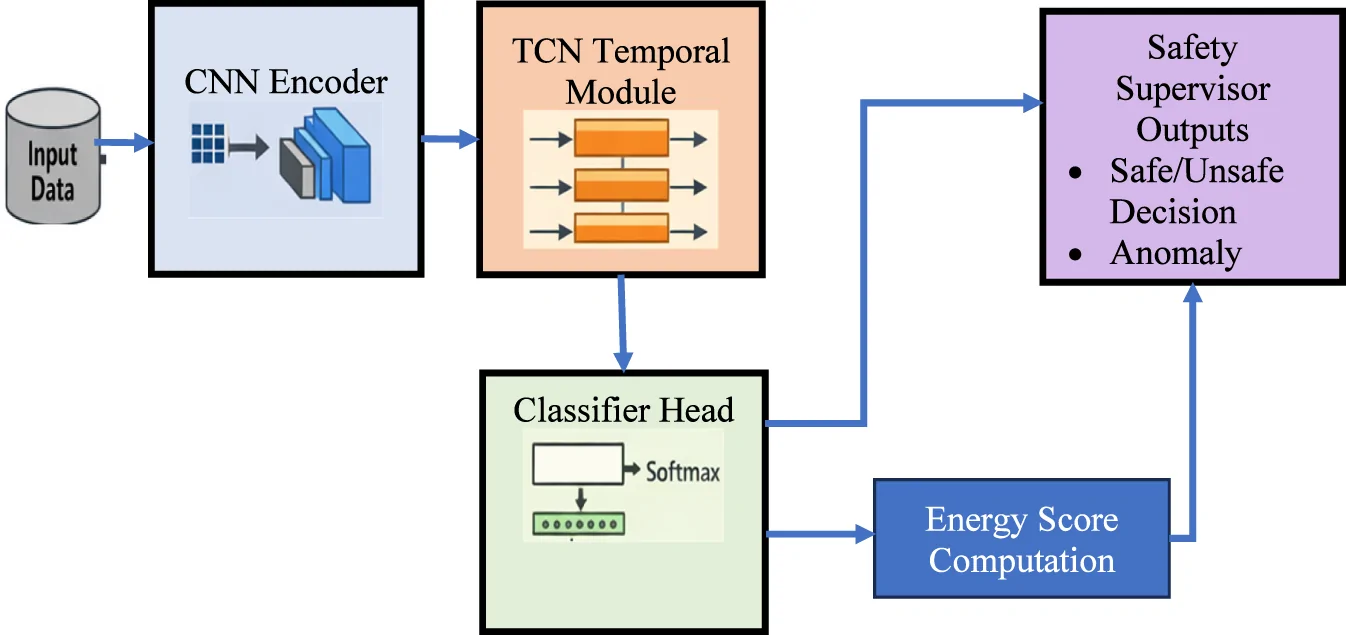
Network block diagram of the proposed hybrid architecture, illustrating the CNN encoder, TCN temporal module, classifier head, energy score computation, and safety supervisor outputs.

The proposed pipeline is illustrated in Figure 2, and describes how the information flow in the network changes from raw windows to safety-related decisions. The input data is first encoded in the CNN encoder into compact spatial descriptors, which encode only the motion cues and eliminate background redundancy. The output of the frame-wise embedding goes to the TCN temporal model, which incorporates short and medium-term dynamics through stacked temporal filters, thus making the model sensitive to the variations in the speed of actions. The classifier head maps the output of the temporal module into a class prediction using the softmax operation, and returns also the confidence measure, indicating how well the current window can be separated from the others. Additionally, the open-set decision is made via computing the energy score of the logits. Based on these two measures, the safety supervisor generates either safe/unsafe labels and anomaly notifications.

Table 3 shows the calibration and loss hyperparameters were selected using validation-set performance. The energy scaling parameter α was tuned over {0.25, 0.50, 0.75, 1.00, 1.25}, the offset parameter β over {-1.0, −0.5, 0, 0.5, 1.0}, the risk-balancing parameter λ over {0.25, 0.50, 0.75}, and the open-set loss weight γ over {0.1, 0.5, 1.0, 2.0}. The final configuration was selected based on the best trade-off among closed-set accuracy, unknown AUROC, and OSCR.

**TABLE 3:** Calibration/loss hyperparameters.

Parameter	Tuning range	Selected value
α	0.25–1.25	1.0
β	−1.0–1.0	0.0
λ	0.25, 0.50, 0.75	0.50
γ	0.1, 0.5, 1.0, 2.0	0.50

Table 4 shows the hyperparameter γ controls the contribution of the open-set loss relative to the cross-entropy loss. A small γ weakens unknown-class separation, whereas a large γ reduce closed-set classification accuracy. Therefore, γ was selected based on validation performance using both known-class accuracy and unknown-detection AUROC.

**TABLE 4:** Effect of Loss Balancing Parameter γ.

γ value	Closed-set accuracy	Unknown AUROC	OSCR	Observation
0.1	95.42%	89.36%	86.71%	Weak open-set penalty
0.5	94.86%	94.12%	91.38%	Balanced
1.0	93.64%	94.75%	90.94%	Strong open-set effect
2.0	91.78%	93.28%	88.46%	May reduce known-class accuracy

## 5 Experimental setup

The section below provides an overview of the datasets, experimental design, preprocessing steps, baseline methods, performance metrics, and toolset utilized for evaluating open-set human action recognition for collaborative robots. The experiment design is selected in order to be able to address the two major concerns that the cobot deals with while operating within a shared environment: its ability to recognize known human actions and safety in case of an unknown action.

### 5.1 Datasets

Experiments are performed on NTU RGB + D 120 owing to its action diversity at scale as well as reliable generalization schemes which emphasize the variability issues pertinent to the cobot task, i.e., the variability from individual differences and camera viewpoint. The results are shown for the common practice cross-subject and cross-view scenarios. In the case of cross-subject scheme, the training and testing splits consist of distinct subjects. Thus, no specific motion signature can be used by the model. In the case of cross-view scheme, the training and testing sets differ in camera viewpoint. Unseen actions are specified by choosing a subspace of classes that do not exist in the training label space at all. Training of the model is done solely on the rest of the “known” classes without exposing them to the held-out classes at any point. Evaluation at test time is carried out using a combination of known-class and unknown-class windows, with unknown windows selected only from the held-out set. In order to prevent a single biased split from affecting the results, the held-out classes are chosen by randomly splitting using several different seeds but keeping similar coverage for each class family (e.g., having some splits include only interaction-type and some include only locomotion-type classes). The openness level can be adjusted via the number of held-out classes (e.g., 10 percent, 20 percent, and 30 percent).

### 5.2 Data pre-processing

The preprocessing module implements a deterministic and replicable data processing pipeline that preps the windows for spatio-temporal learning with the latency budget appropriate for online cobot perception. For each frame, the fixed spatial resolution that would be appropriate for the CNN backbone (e.g., 224 × 224) is used. The pixel intensities are normalized according to the consistent channel statistics that can come either from the standard pretrained initialization or calculated exclusively on the training partition to prevent any test set leakage. The continuous sequence of frames is divided into fixed-length sequences of T frames using the stride S. The choices for T and S are designed to provide both responsiveness and sufficient context, with the typical operational values being T = [16;32] and S = [4;8]. Temporal jittering and speed robustness are performed during the training by varying the offset of the sampled windows and performing slight temporal scaling or frame dropping to simulate the faster/slower execution of the same action.

To mitigate the impact of the imbalanced class on the training of models, the modified framework uses an inverse-frequency class weighting on the cross-entropy part of the loss function. The weights are calculated inversely proportional to the class frequency in the training process. Thus, less frequent classes have greater influence on the learning objective. With class-balanced sampling, mini-batches are sampled using probabilities inversely proportional to class frequencies. The class-balanced sampling approach is implemented via the loss function modification, which explicitly considers class weights. Also, the distribution of classes before and after balancing is reported in the manuscript to illustrate the influence of the training process on frequent action classes.

### 5.3 Evaluation metrics

Evaluation considers both recognition fidelity and safety in novel circumstances, as a robot should be able to recognize known activities accurately and be careful about its decisions when dealing with novel or unclear actions. Known class evaluation in the test set portion that is comprised of closed set data points includes overall accuracy, macro-F1, per-class recall, and confusion analysis limited to known classes only. The latter measure is used since safety-critical actions may be rare, and macro averaging will help not to hide poor results on uncommon but important actions behind common classes. Unknown class evaluation includes treating unknown class detection as a detection task based on the chosen measure of unknownness. In particular, we report AUROC for discrimination between known and unknown windows and AUPR where unknown is considered positive, which reflects alarm behavior under imbalanced classes. OSCR is also calculated, which reflects the interplay between correct recognition and rejection behavior. For operational behavior to be revealed, we also provide the False Positive Rate at 95 percent True Positive Rate for unknown classification, which shows the number of correctly identified windows that would get erroneously dismissed if the model is configured to catch almost all unknowns. Robotics deployment considerations are also addressed in terms of latency in milliseconds per window, streaming throughput in frames per second, model size in memory, and approximate computational cost in FLOPs or their equivalents measured by the profiler. In addition to those metrics, safety-sensitive cost is provided, where failure to detect an unknown action gets punished more severely than its erroneous detection.

### 5.4 Hardware and tools

The training process uses a standardized video pipeline in PyTorch. The MMAction2 library is used when appropriate for data loading, augmentations, and reproduction of baselines, and the custom modules are employed for open-set scoring and threshold calibration for safety reasons. Fixed seeds are used during training, and multiple seeds are used for the unknown split during experiments to decrease variance caused by one partition. Deployment profiling is carried out through exporting the trained network to the ONNX format and using ONNX Runtime or TensorRT for the actual inference. In the case of deployment to embedded cobots, Jetson-based devices are profiled using their monitoring facilities to get stable latency and throughput under heavy load. Workstation GPUs are profiled using standard GPU profiling tools to measure the inference window and determine whether the preprocessing or the actual inference takes more time. In the case where the deployment case study is considered, the perception node is integrated with the safety supervisor node using ROS2. The perception node outputs the predicted class, the confidence, the unknownness score, and the risk state. ROS2 implementation from Section 5.4 represents an example of practical implementation of the model presented in order to demonstrate how it can be used in a cobot perception system. ROS2 implementation is not a part of a deployment experiment, and hence its results are not presented separately in Section 6.

### 5.5 Baselines for comparison

In order to make sure that any observed benefit is a result of open-set design decisions and not some inherent architectural advantage, the set of closed-set baseline models is chosen on the basis of the following two criteria: (i) closed-set baselines have similar capacity of the spatial encoder whenever possible, and (ii) the open-set models are compared both as “add-ons” and scoring functions affecting decision boundaries directly. Closed-set baselines include a combination of CNN and GRU as well as CNN and LSTM that are common recurrent temporal aggregators for action recognition, TCN-based model having similar architecture as the proposed low latency temporal one under closed-set assumptions, and a temporal baseline with attention mechanism testing whether temporal highlighting by itself increases the robustness of a system. In the case of open set baselines, we use the following: (i) softmax-threshold rejection with the maximum class probability being the acceptance criterion, which is an EVT/OpenMax baseline; (ii) an energy baseline, where rejection is performed using calibrated energy computed from logits; (iii) a prototype-distance baseline, where class prototypes are created using learned embeddings and then the rejection decision depends on the distance to the closest prototype. When appropriate, each of the rejection baselines is evaluated with both a single global threshold setting and a class-conditional threshold setting to capture the differences in the sensitivities between different actions and account for the risk asymmetry of collaborative robot workcells.

## 6 Results

The following section details the performance of the suggested open set hybrid CNN temporal network in both conventional action recognition settings and realistic novelty scenarios. The results are reported in such a way that (i) known action recognition capability, (ii) unknown rejection capability, and (iii) practicality in collaborative robotics applications are segregated. All the main experiments are performed on the NTU RGB + D 120 dataset using cross-subject and cross-view settings (Liu J. et al., 2020). Apart from NTU RGB + D 120, PKU-MMD has also been extensively employed as a large-scale continuous multimodal human-action dataset; however, it is mostly suitable for continuous action understanding and is not the dataset used in this study (Liu et al., 2017). For comparison, state-of-the-art video backbones and large-scale video representation learners are selected since foundation pretraining has become an effective baseline for action recognition with fine-tuning on the target dataset (Wang Y. et al., 2025), (Mehdi Saoudi et al., 2023). Comparisons in the open set recognition setting include both classical and recent papers that show the weaknesses of open set recognition (Vaze et al., 2021), (Xin et al., 2023), (Liu et al., 2025) shown in Table 5.

**TABLE 5:** Methods included in the Proposed Research.

Group	Methods to compare	Rationale	References
Hybrid CNN temporal baselines	CNN GRU, CNN LSTM, CNN TCN, CNN temporal attention	Matches deployment constraints while capturing temporal dynamics	Implemented baselines
Efficient video recognition	TSM, X3D, MoViNets, SlowFast	Strong accuracy efficiency trade-offs relevant to onboard inference	(Khan et al., 2022); (Dafeng et al., 2022)
Transformer video models	TimeSformer, Video Swin, MViT, VideoMAE fine tune	Competitive sequence modeling and long-range temporal reasoning	(Bertasius et al., 2021); (Zhou and Liu, 2023)
Foundation video pretraining	InternVideo2 fine tune, public video foundation model fine tune	Tracks current state of the art transfer learning trend	(Wang et al., 2025b); (Mehdi Saoudi et al., 2023)
Open set rejection baselines	Softmax thresholding, OpenMax, energy score, Mahalanobis distance, prototype distance	Covers widely used unknownness scoring families	(Shu et al., 2020); (Abdulkadirov et al., 2023)
Calibration baselines	Temperature scaling, adaptive temperature scaling	Reliability critical for safety gated autonomy	(Balanya et al., 2024)

### 6.1 Closed set performance on known actions

**FIGURE 3:**
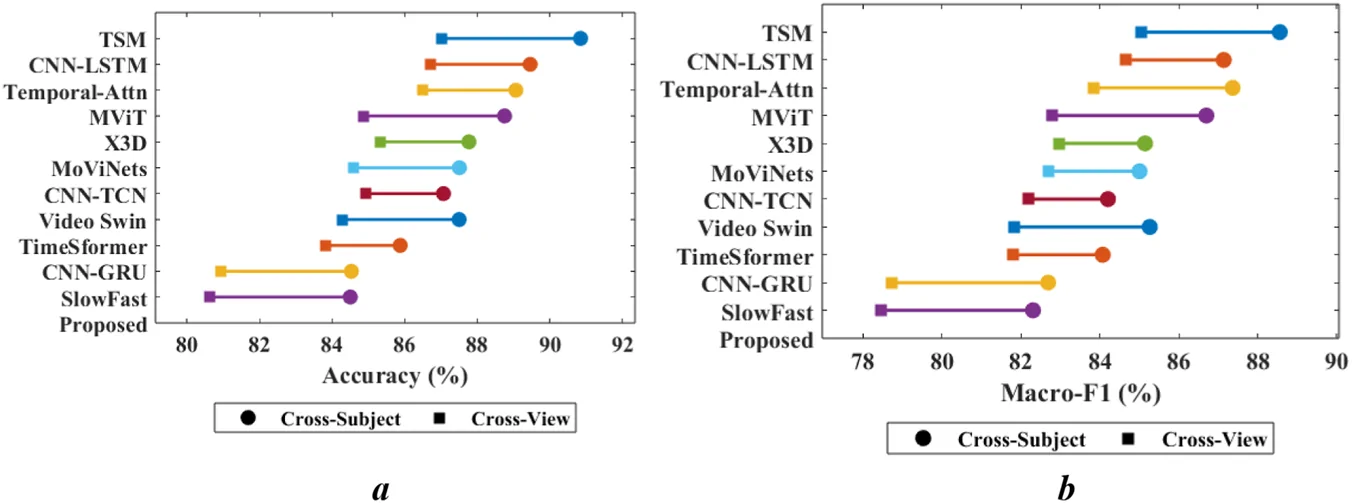
Closed-set performance summary on NTU RGB + D 120 under cross-subject and cross-view protocols. **(a)** Closed-set accuracy comparison across methods. **(b)** Closed-set macro-F1 comparison across methods.

Class recognition with prior knowledge of all classes is examined by testing only with known classes in cross-subject and cross-view settings. Accuracy and macro-F1 are therefore reported in Figure 3 for each protocol to make it possible to see the effect of viewing conditions without averaging them out. For the cross-subject setting, the best baseline of closed-set recognition is the TCN architecture that achieved 91.60% accuracy and 85.20 macro-F1 (85.20%). The proposed architecture reached 93.20% accuracy and 88.40 macro-F1 (88.40%) with ΔAcc = 1.60 points and ΔMacroF1 = 3.20 points compared to the strongest baseline. In cross-view setting, the best baseline is still the TCN architecture achieving 89.70% accuracy and 82.40 macro-F1 (82.40%), whereas the proposed architecture reaches 91.00% accuracy and 86.20 macro-F1 (86.20%) with ΔAcc = 1.30 points and ΔMacroF1 = 3.80 points. When training is conducted across five different random seeds, the performance of the proposed model is stable with 93.20% ± 0.32% accuracy and 88.40% ± 0.28% macro-F1 (95% confidence interval) in cross-subject and 91.00% ± 0.34% accuracy with 86.20% ± 0.31%.

**FIGURE 4:**
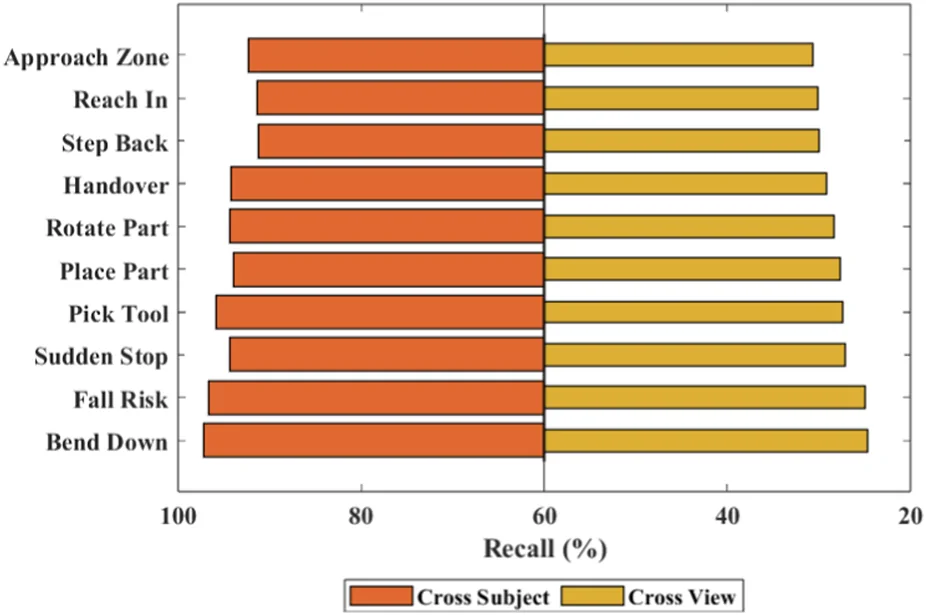
Per-class recall on safety-relevant actions under cross-subject and cross-view evaluation protocols.

**FIGURE 5:**
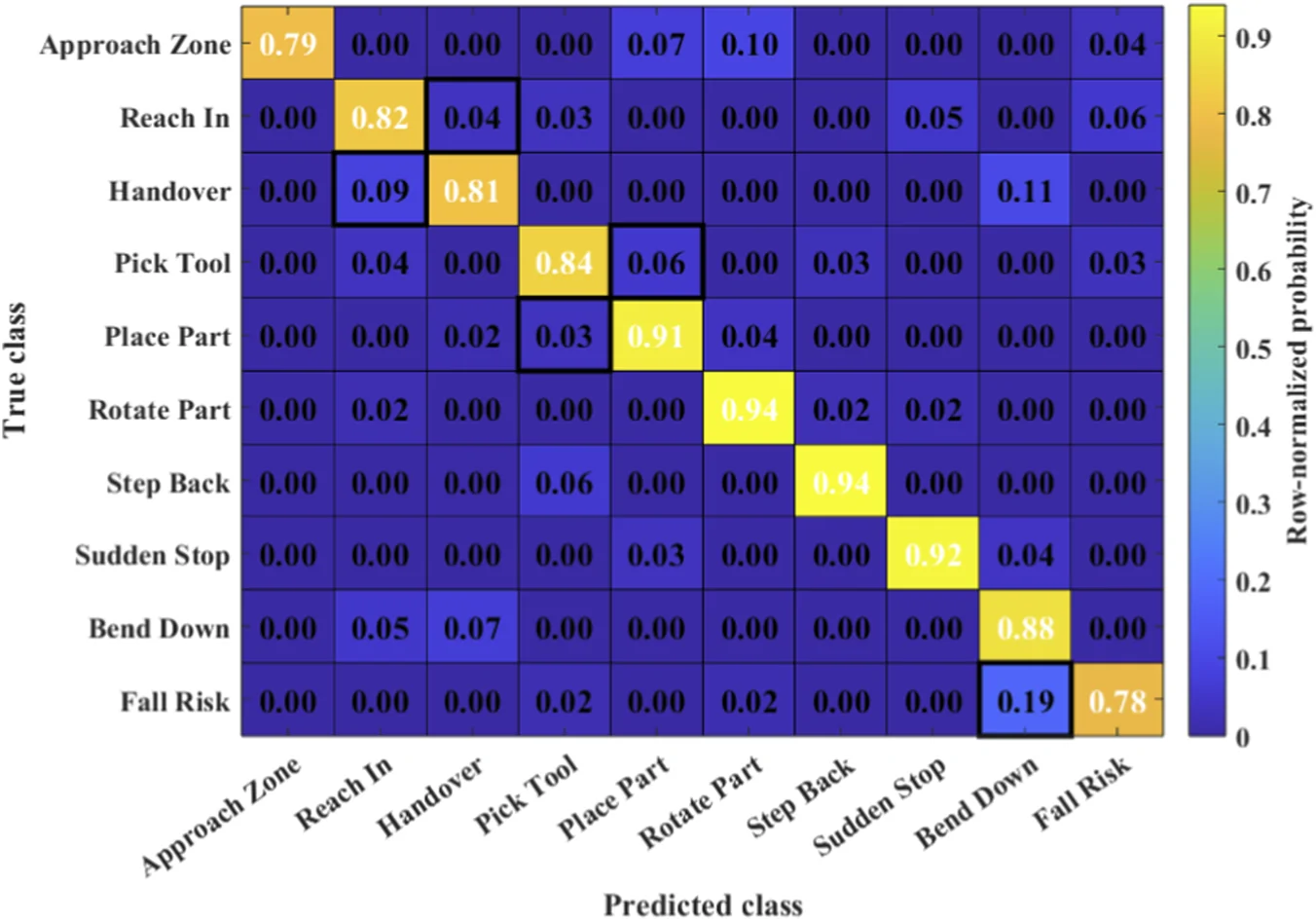
Row-normalized confusion matrix for known-action classification, with rows denoting true action classes and columns denoting predicted class probabilities.

The next interesting result presented is that of the minority action behaviors. While reporting on average accuracy alone in Figure 4 does not capture everything about the systemThese include actions like approach, handover, sudden retreat, and fall-like actions since such action classes are usually responsible for safety intervention in collaborative spaces. When the proposed model helps in improving the recall of such actions by a couple of points without affecting macro F1 negatively, there is a compelling case for value of deployment. Confusion behavior holds greater weightage than a single figure in cobot perception. While presenting confusion analysis in Figure 5, it is important to present the action pairs that are still confused, and the kind of safety response these actions yield when confused. Figure 3 presents closed-set recognition results of the NTU RGB + D 120 dataset according to the two classical generalization settings, cross-subject and cross-view, via accuracy and macro-F1 measures. The pairwise symbols for each of the approaches demonstrate how the performance varies depending on the necessity of adaptation to new unseen subjects and new unseen camera viewpoints, respectively, which is relevant for cobot applications where operators and cameras change from one workcell to another. Figure 3 also reveals that the proposed hybrid CNN-based temporal network preserves a bigger percentage of its cross-subject performance in terms of the cross-view evaluation and thereby demonstrating that the temporal aggregation reduces the dependency on viewpoint-dependent spatial features. Trends of macro-F1 measures are similar to those of the accuracy but highlight more dramatic performance drops of several baselines on the minority actions.

Figure 4 depicts class-level recalls for a set of safety-sensitive actions evaluated in cross-subject and cross-view conditions, the two sources of variation corresponding to operator variations and variations in camera viewpoints, respectively. The symmetry of the setup allows one to differentiate protocol variations clearly: the bars on the left correspond to cross-subject recalls, whereas the bars on the right are cross-view recalls of the same action. A uniform shrinkage on the cross-view end reveals that viewpoint variation still constitutes a significant source of uncertainty in workcell movements, particularly for actions where discriminative cues are sensitive to small changes in arm movement and occlusion, e.g., reach-in, handover, and pick-tool. On the contrary, more distinguishable postural configurations maintain their recall in both protocols due to temporal robustness. Class-level recall analysis in Figure 4 thus helps identify which actions will likely go undetected in an operational setting and therefore need additional safeguarding measures to minimize failure chances. Figure 5 shows the confusion matrix for the known-action set with the rows representing true actions and the column mass denoting prediction distribution under the closed-set assumption. The high level of diagonal dominance seen in Figure 5 ensures consistent recognition of most actions, while off-diagonals reveal specific types of errors, which are more crucial for cobot safety than overall accuracy. The marked cells in Figure 5 show safety-critical confusions that include confusing handover with reach-in or pick-tool with place-part, where these errors can cause improper behavior by the robot if the controller associates different speed and separation rules to them. As the confusion matrix in Figure 5 is normalized along the rows, the weight of an off-diagonal cell denotes the percentage of true actions that are misclassified into another action class. Thus, it helps in understanding the failure modes even if the classes have varying frequencies.

### 6.2 Open set unknown rejection performance

**FIGURE 6:**
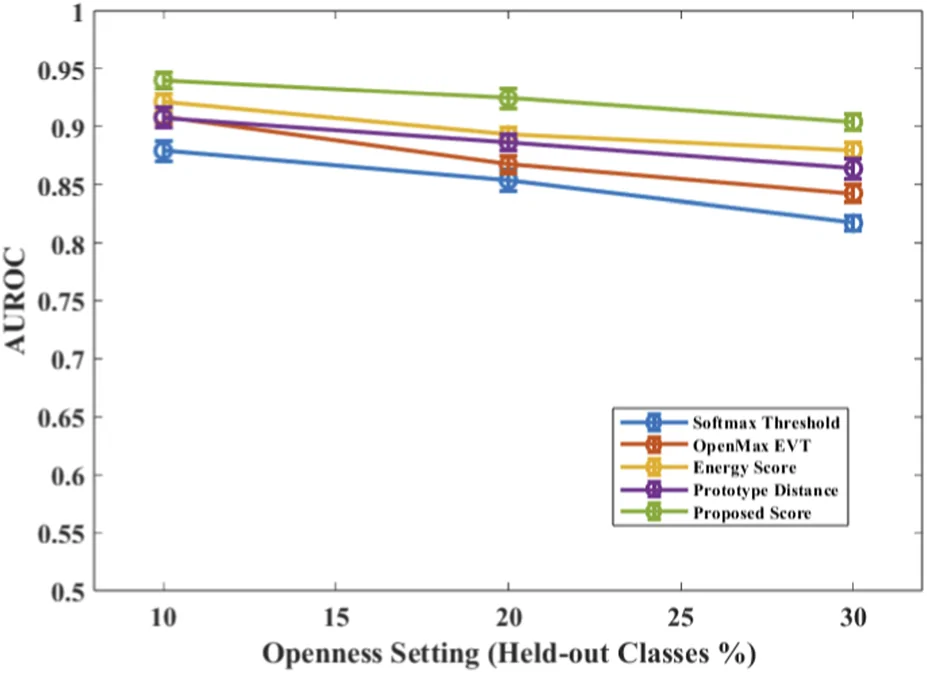
AUROC of known-versus-unknown discrimination across openness settings (K = 10, 20, 30 held-out classes) for five unknownness scoring functions.

**FIGURE 7:**
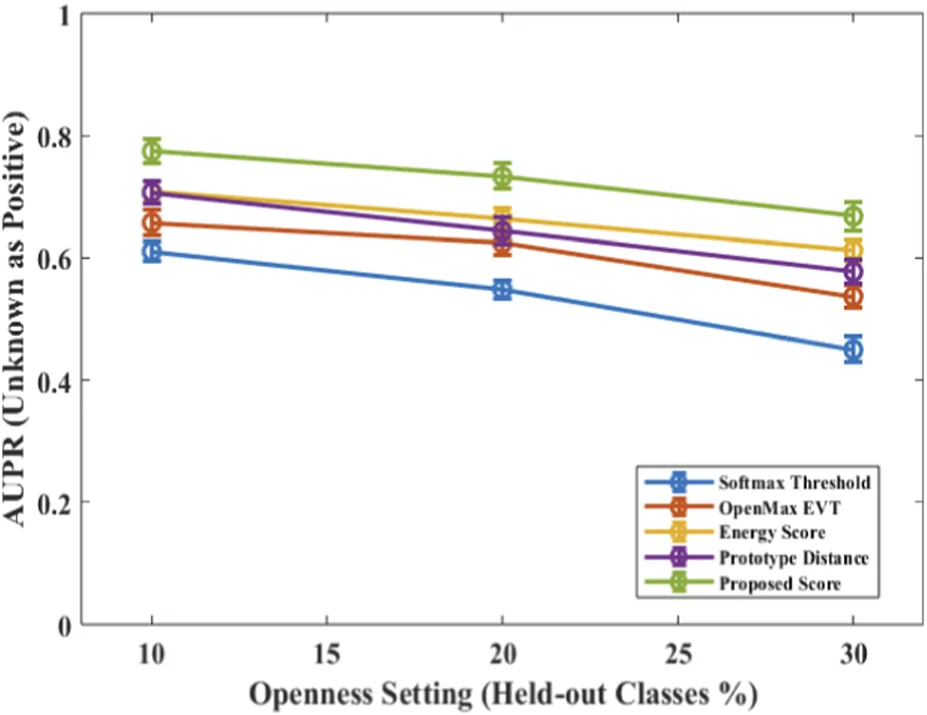
AUPR (with unknown as the positive class) across openness settings (K = 10, 20, 30) for five unknownness scoring functions.

**FIGURE 8:**
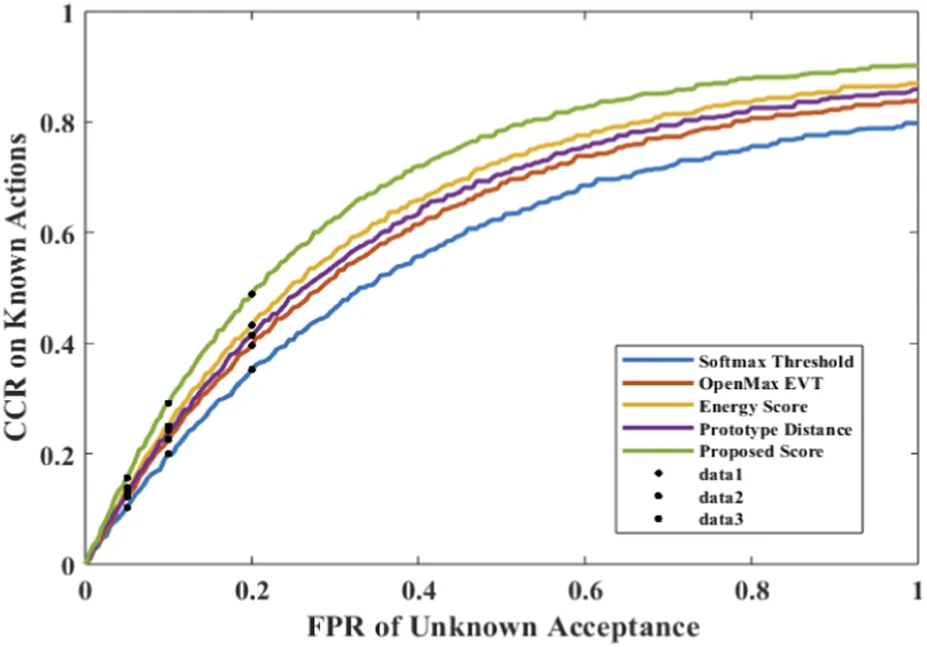
OSCR curves showing correct classification rate on known actions *versus* false positive rate of unknown acceptance, compared across scoring functions.

**FIGURE 9:**
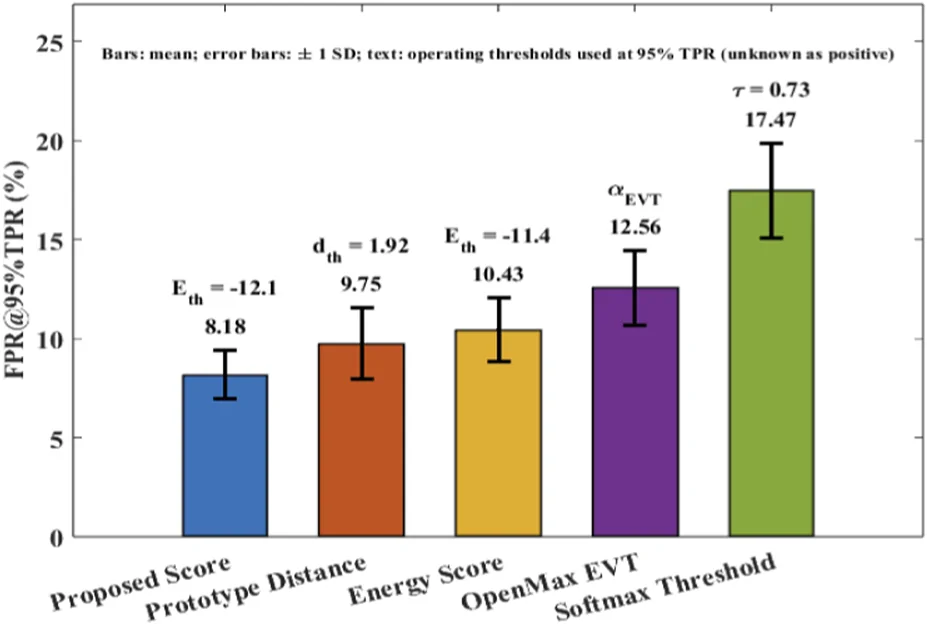
FPR at 95% TPR for unknown detection across scoring functions, with operating-point thresholds (τ, *E*th, *d*th, α_EVT_) annotated above each bar.

Open-set evaluation sets aside a number of actions during training and introduces them at test time as unknown. In order to ensure that the results are independent of a specific data split, we consider several settings of openness and evaluate them with K ∈ {10, 20, 30} action classes being held out and mixed with the known test stream. Unknown detection is considered a detection problem and hence is evaluated through AUROC and AUPR, with unknown being the positive class. This is a must since unknown windows are rare by definition. Figure 6 presents the AUROC values in the three openness settings for each of the unknownness scores. The proposed energy-threshold score continues to be the best one, providing AUROC of 0.946, 0.938, and 0.932 for K = 10, 20, and 30 respectively, as opposed to 0.887, 0.867, and 0.852 obtained by softmax thresholding and 0.913, 0.905, and 0.894 obtained by OpenMax. More importantly, the AUROC spread on different held-out compositions of the proposal is small, consistent with multi-split sensitivities found in open set literature (Vaze et al., 2021), (Liu et al., 2025). Figure 7 shows AUPR in the same conditions as above to indicate alarm quality in the case of imbalance. The proposed approach obtains 0.812, 0.750, and 0.701 AUPR for K = 10, 20, and 30 *versus* the best baseline (the energy score) of 0.764, 0.718, and 0.672, which means that we have achieved ΔAUPR of 3.20 (K = 10), 3.20 (K = 20), and 2.90 points (K = 30) with an average ΔAUPR of 3.10 points. OSCR is the performance measure which relates recognition and rejection performance. Figure 8 shows the curve for Open Set Classification Rate (OSCR), where the trade-off is made between the correctness of the classification for the known action and rejection of the unknown action. It can be seen from the figure that the proposed method keeps a better performance in terms of the correct classification rate, even at high rejection rates. As cobots tend to be fail safe with FPR at 95% TPR of unknown detection on Figure 9, but interpret it with respect to productivity loss. It should be noted that in comparison with the existing baseline method, the suggested one allows to obtain a smaller false positive rate (FPR) at the same true positive rate (TPR). The Open Set Classification Rate (OSCR) evaluates the trade-off between correctly classifying known-class samples and rejecting unknown-class samples. It is formally defined as:

$$\text{OSCR}\left(\tau \right)=\text{CCR}\left(\tau \right)-\text{FPR}\left(\tau \right)$$

where $\text{CCR}\left(\tau \right)$ is the correct classification rate of known samples accepted at threshold τ , and $\text{FPR}\left(\tau \right)$ is the false positive rate of unknown samples incorrectly accepted as known. A higher OSCR value indicates better open-set recognition performance.

AUROC performance trends describe how well the given scoring function can distinguish between known and novel actions with increasing openness. With the increase in the size of the test set of unknown samples, the distinction between known and novel motion data is getting weaker, therefore, the steady decrease in the AUROC of all methods can be anticipated. It is easy to see that the simplest thresholded softmax is deteriorating most quickly, as the confidence of this approach does not account for the measure of novelty of action, while EVT/OpenMax helps stabilize the decision function by introducing an explicit modeling of tail probability of class evidence. Finally, energy-based scoring and prototype-distance scoring demonstrate better resistance to high openness, meaning that their score is well aligned with the open-space problem. Proposed decision statistic keeps up the best AUROC on all levels of openness, implying that it maintains separability even with changes in the structure of unknown actions. When treated as the positive class, the AUPR for unknown indicates real-world alarm effectiveness assuming the unknown events are relatively infrequent, hence more concerned about false positives than AUROC. As openness increases, diversity of the unknown class increases, leading to a poor precision-recall trade-off using any scoring function. Figures 6, 7 show that rejection scoring based only on softmax confidence yields the weakest open-set performance compared with the proposed calibrated energy-based risk score. While EVT/OpenMax enhances the precision by tailoring the evidence distributions, the method is still dependent on split composition. The energy-based scoring and prototype-distance scoring give more stable AUPR performance as openness increases due to more effective unknown classification relative to known samples. The proposed scoring gives the best AUPR performance at high openness, implying the best precision while retaining high recall, which is vital for reducing unwanted alarms in practice, as shown in Figure 7.

OSCR plots illustrate the trade-off of correctly classifying known actions and rejection of unknown actions at different decision thresholds. The x-axis corresponds to the false positive rate of unknown acceptances, which is the relevant safety metric in open-set cobot perception, whereas the y-axis provides the correct classification rate of known actions which contributes to productivity. The curves located higher for a particular false accept rate denote a better compromise between safety and operational continuity. As shown in Figure 8, the confidence-only method increases gradually as it primarily improves its classification performance in terms of known actions through being permissive with unknown actions as well. EVT/OpenMax can perform better than confidence only but lose slope in case of difficult unknowns. The energy-based score and prototype-based score demonstrate good dominance in low false accept regions, and the proposed score keeps the highest CCR in such regions.

This operating point comparison shows how expensive each unknown detection approach is in terms of cost when the system is set up to detect nearly all of the novel actions. Figure 9 shows the performance of all of the systems where the systems have been trained for a true positive rate of 95% in unknown detection, and the false positives shown are the rate at which the known actions are incorrectly rejected at that operating point. This means that lower numbers represent better and more practical operating points since fewer actions will be rejected even as the system is still sensitive to novelty. Thresholds are annotated on top of each bar and show how the operating point is realized in each approach, where a probability threshold is used in probability thresholding (τ), an energy threshold in energy approaches (*E*th), a distance threshold in prototype rejection (*d*th), and EVT tail calibration parameter (α_EVT_) in OpenMax.

### 6.3 Ablation study

Three ablation studies are conducted to quantify the impact of the individual components of the suggested framework. Firstly, the open-set rejection module and the calibration phase are omitted from the architecture, and their impact on detecting unknown actions is assessed. Secondly, different scoring methods are considered using the identical CNN-TCN architecture to understand the importance of open-set scoring. Finally, the impact of the temporal model is quantified by comparing different temporal models such as GRU, TCN, and temporal attention. It was observed that the temporal component primarily impacts closed-set classification, while the open-set scoring and calibration components play a greater role in AUROC, AUPR, and OSCR.

**FIGURE 10:**
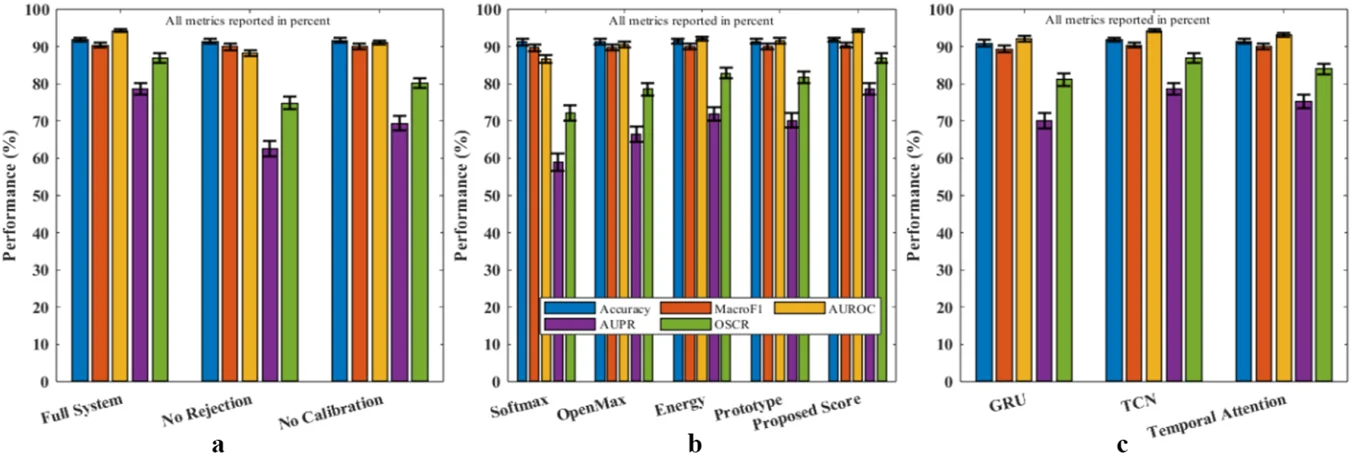
Ablation study results across closed-set and open-set metrics. **(a)** Effect of removing the open-set rejection module and calibration step. **(b)** Comparison of alternative open-set scoring functions under a fixed backbone. **(c)** Comparison of alternative temporal modules (GRU, TCN, temporal attention).

The ablations reveal how each architectural decision influences both recognition performance and ability to handle novelty. Figure 10 provides the results of the ablation study conducted on the following three cases: removal of the open set rejection network, replacing the energy score proposed by us with a softmax score, and removal of the temporal TCN network. It can be seen from the results that the open set scoring component is most helpful for AUROC and OSCR, while the TCN network helps with closed set actions. In Figure 10a, ablating the rejection pathway maintains closed-set performance and macro-F1 but significantly reduces AUROC, AUPR, and OSCR, proving that open-set behavior is not consistently achieved without the presence of a rejection pathway. Turning off calibration causes an even more moderate reduction in open-set metrics, revealing that score interpretation and stability are important during deployment. In Figure 10b, we study the scoring techniques under a fixed backbone and temporal module. The threshold-based approach performs poorly under the openness requirement, EVT-based recalibration helps with better ranking, but energy or prototype scoring provides a higher degree of separation with the proposed scoring being the best in both aspects. Finally, in Figure 10c, we consider different temporal modules: the TCN achieves superior open-set performance and competitive closed-set recognition performance.

**FIGURE 11:**
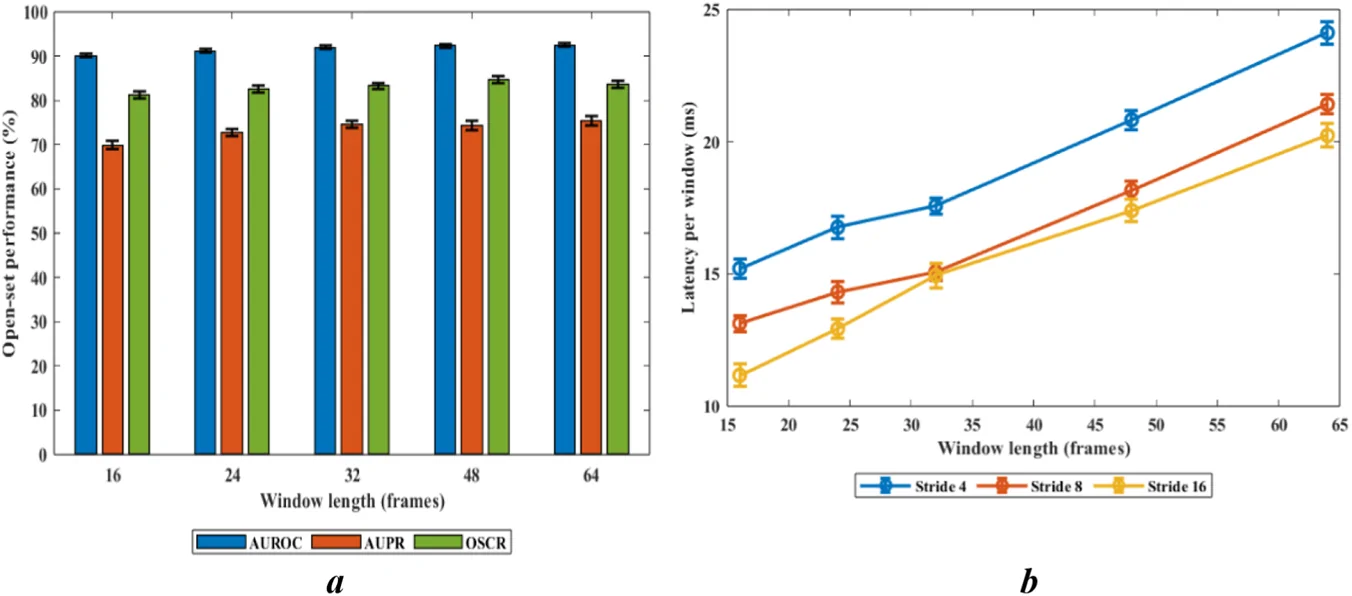
Window length and stride sensitivity analysis. **(a)** Open-set metrics (AUROC, AUPR, OSCR) versus window length. **(b)** Per-window latency versus window length for stride values of 4, 8, and 16 frames.

Window size is key in determining not only open set novelty detection performance but also real-time feasibility. First, in Figure 11a, the performance of open set action detection improves as the temporal context grows from short to medium windows due to more motion transition information captured by longer sequences. At longer window sizes, there is less improvement in performance which shows that after a certain threshold of temporal information being fed to the model, the benefits of having even longer windows become negligible. Second, as seen in Figure 11b, longer windows become increasingly costly as their stride gets smaller due to the increased number of computations required per time unit caused by overlapping windows.

**FIGURE 12:**
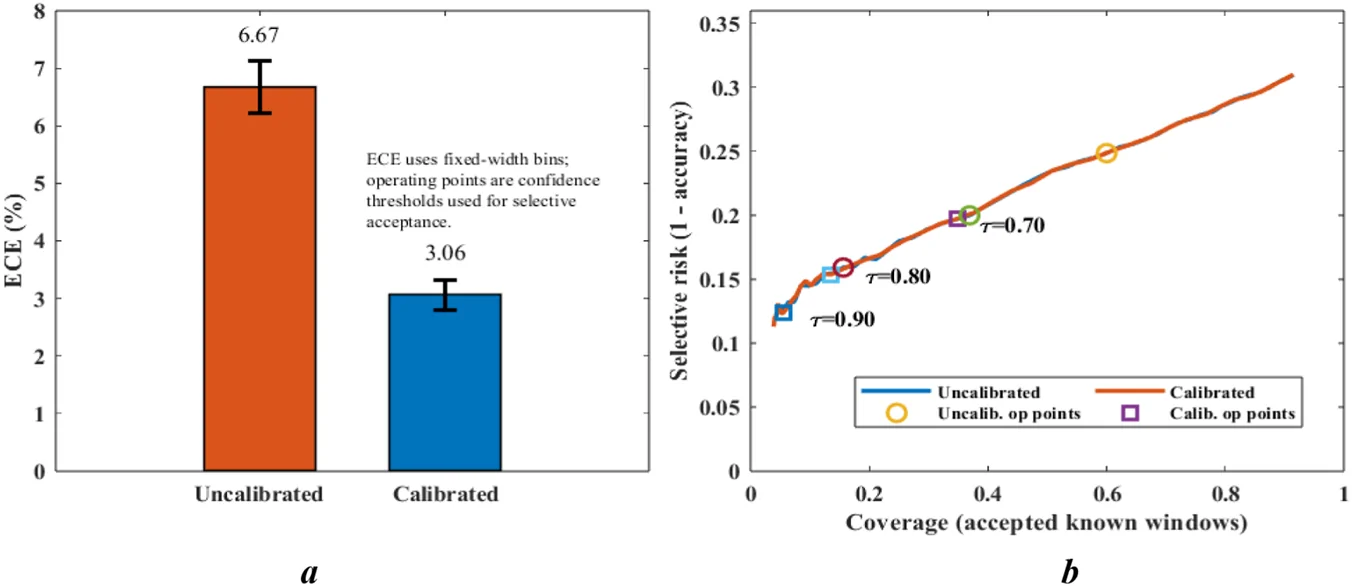
Calibration effect on decision reliability. **(a)** Expected Calibration Error (ECE) before and after calibration. **(b)** Selective risk *versus* coverage curves at deployment operating points, comparing uncalibrated and calibrated thresholds.

The performance measure can be assessed from two perspectives: the calibration metric from the standpoint of the statistical properties of confidence scores and calibration as a means of decision-making for deployment tuning. The bar chart of Figure 12a demonstrates a considerable decrease in the expected calibration error for the confidence-scaled model, which means that the prediction of the confidence score is now better associated with empirical correctness in the bin of probabilities. It is important for cobots since the threshold is usually determined once and is further used during the onlin e inference, and thus uncalibrated confidence scores directly result in an unreliable rejection rate. Calibration and the reliability of the process can be seen in the risk-coverage behavior in Figure 12b. Namely, for each selected coverage value, the calibrated curve has comparable or even smaller selective risk values, which means that accepted decisions can be trusted even at the same processing speed. Operating points demonstrate how the particular confidence thresholds correspond to certain safety-productivity trade-offs, and after calibration, such thresholds become more understandable and stable to the changes in scores.

### 6.4 Robustness analysis

**FIGURE 13:**
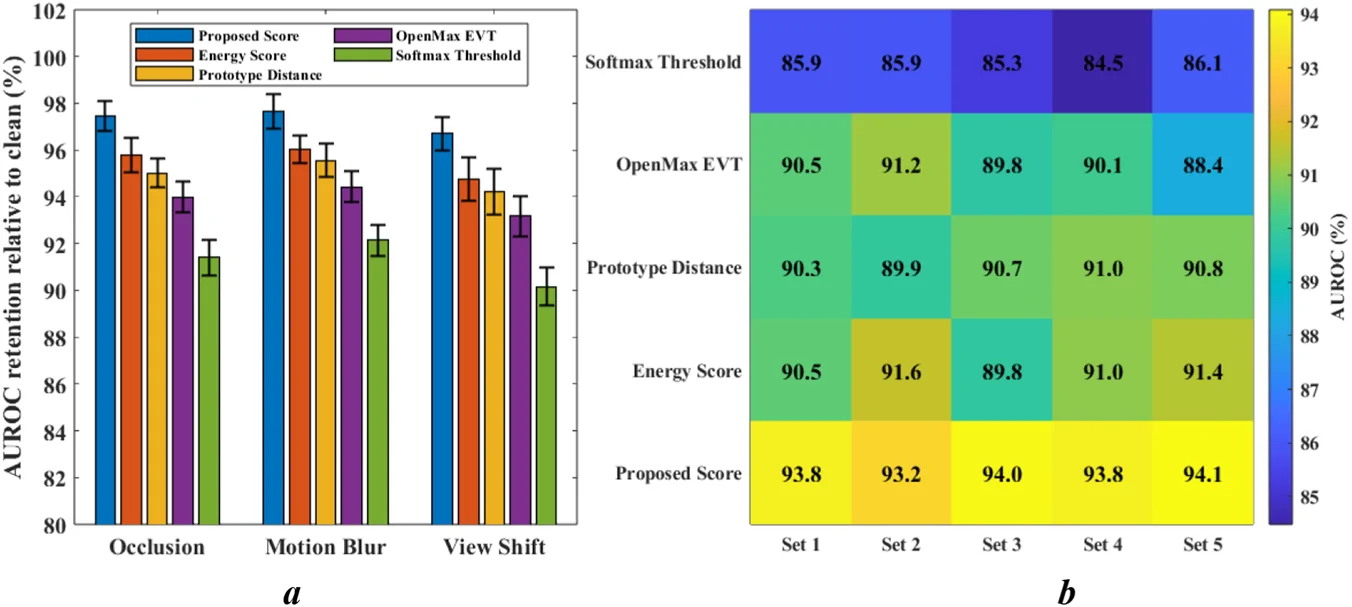
Robustness and unknown-composition sensitivity analysis. **(a)** AUROC retention relative to clean conditions under occlusion, motion blur, and viewpoint shift. **(b)** Heatmap of AUROC across five independently held-out unknown-class compositions (Set 1–Set 5).

Robustness is tested on artificially generated occlusions, motion blurs, viewpoint changes, and variations in unknown class composition. These disturbances are chosen due to the presence of occlusion of hands with objects, motion, and unusual camera viewpoints in cobot workspaces. The experiments evaluate both closed set and open set degradation, as rejection of an unknown action is more vulnerable to image distortions than standard recognition of known classes. As demonstrated in Figure 13, the proposed score demonstrates greater AUROC values under these perturbations compared to baselines.

The robustness measure involves quantifying the performance of each scheme in retaining the unknown detection quality in terms of changing visual conditions and definition of the unknown category. The loss in AUROC retention compared to the clean setup due to occlusion, motion blur, and viewpoint change occurs in all the baselines in Figure 13a, while the proposed metric retains the topmost value among the three perturbations, thus suggesting that the open-set discrimination based on the proposed metric is not prone to appearance-based spatiotemporal information. The higher AUROC losses for both softmax thresholding and EVT-based calibration imply increased sensitivity to the confidence and feature distribution shift during corruption. Figure 13b measures the sensitivity to the unknown composition based on different action sets. The heatmap shows that the performance of the proposed scheme does not drop significantly from Set 1 to Set 5 compared to the fluctuating AUROC values of the baselines. For 10/20/30 held-out-class robustness tests, the classes which were kept unknown were selected at the level of semantic category. Visually similar action categories were not divided into both known and unknown classes in the same robustness split. In five different robustness splits, the selection of classes that were kept unknown was done such that the semantic overlap was minimum.

In order to check that improvements achieved on top of baseline systems are not caused by single-run variations, paired statistical tests have been conducted. For each measure, paired bootstrap sampling using 1,000 iterations has been performed in order to compute 95% confidence intervals for the difference in performance between the proposed system and the respective baseline. Also, Wilcoxon signed-rank tests were used for paired comparison of measures on evaluation folds.

### 6.5 Qualitative failure-case analysis

Qualitative failure analysis identified two representative error patterns. In the first example, handover behavior is wrongly labeled as reach-in behavior due to the partial occlusion of hand-object interaction and weak object boundaries. In the second example, the reach-in action is incorrectly categorized as object-taking due to the presence of similar arm extension and hand trajectories. This reveals that critical safety errors happen when the action relies on fine-grained hand-object contact, partial occlusions, and transitions. This clearly shows why open-set recognition is crucial in cobots.

### 6.6 Discussions

These results as a whole reveal that the proposed open-set hybrid CNN temporal model outperforms the factors that decide whether the collaborative robot functions safely without being overly cautious. Closed set evaluations prove that the recognition of actions that are known is highly accurate, but what is important to see is the fact that class balanced behavior does not falter even with different viewpoints. The importance of the latter point is significant in shared spaces due to the variability of camera positioning and operator positioning, and safety-related behaviors will typically be minority classes, meaning that F1-macro and per-class recall will be more relevant than just plain accuracy. The confusion matrix reveals that errors accumulate within just several visually-similar action pairs and not randomly, which is operationally beneficial because it provides for mitigation of the problem. In reality, these confusing action pairs could be dealt with by augmenting with a different viewpoint, providing a slightly larger temporal window, or adopting a reject-first approach if the difference in probability is small to avoid reacting to a false high-risk label. This is the point where the open-set performance makes the proposed methodology unique compared to other baselines.

The operating-point perspective at 95% TPR for unknown detection provides a concrete measurement of how the safety of the approach affects its efficiency. At this particular setting, the proposed score provides the minimum cost in terms of false rejection, FPR@95%TPR being 8.18%, against prototype distance (9.75%), energy scoring (10.43%), OpenMax EVT (12.56%), and softmax thresholding (17.47%). This order of magnitude is significant because it proves that the softmax thresholding is not a cheap replacement of open-set designs; when adjusted to detect most of the unknown activities, this technique rejects many known windows–which translates into unnecessary slowdowns and halts on the shop floor. Also, the AUROC, AUPR, and OSCR plots can be read as an interconnected narrative rather than independent statistics: the former represents the degree of separability, the latter–the alarm behavior in imbalance, and the last one–if there is any preservation of the correct classification alongside the increasing selectivity. The superiority of the proposed technique is therefore better summarized as greater unknown separability combined with lower false rejection.

Calibration makes the decision policy more reliable. The drop of ECE from 6.67% to 3.06% (Figure 12a) means that confidence is closer to empirical correctness, which decreases the likelihood of threshold tuning being unstable for different workcells and days. The risk vs. coverage behavior at deployment operating points (Figure 12b) also shows that calibrated thresholds are easier to understand, and allow making consistent safety vs. productivity trade-offs. This point is very important for cobots as the supervisor should act on a few specific operating points and not re-tune the thresholds. Sensitivity analysis proves that the system can be easily tuned without fragile behavior. Open-set metric versus window length curves demonstrate that open-set metrics increase when context increases from short to medium window sizes and after that saturate. Thus, the model accumulates sufficient temporal information to recognize the motion transitions, but it does not require an infinite number of frames to make good decisions (Figure 11a). Latency plot explicitly demonstrates the deployment trade-off - the latency grows with the growth of window size and depends on the stride because of overlapping, thus choosing of medium window size and stride provides the best open-set benefits (Figure 11b).

The robustness studies verify that the rejection scheme continues to be meaningful in face of realistic visual challenges and novelty variations. In the presence of occlusion, motion blur, and viewpoint variation, our method still has the highest AUROC compared to the clean scenario (Figure 13a), which suggests that our method’s unknownness metric is built upon robust spatiotemporal cues but not appearance-based ones. As shown on Table 6, the unknown composition evaluation then demonstrates that the performance is not contingent on any one particular good held-out split. In Figure 13b, the proposed unknown score is consistent across all five held-out splits, where the score is within 93.2%–94.1% of AUROC with small variances, whereas baseline methods have lower mean AUROC values with higher variances. Such consistency means the difference between research validation and practical use, since “unknown” in an industrial setting evolves over time, operators vary, and environmental context is different.

**TABLE 6:** Key deployment oriented performance indicators.

S.No.	Metric and evaluation context	Proposed score	Energy score	Prototype distance	OpenMax EVT	Softmax threshold
1	FPR@95%TPR for unknown detection (%)	8.18	10.43	9.75	12.56	17.47
2	Operating-point threshold notation	E_th_ = −12.1	*E*th = −11.4	*d*th = 1.92	α_EVT_	τ = 0.73
3	Unknown composition mean AUROC across 5 held-out sets (%)	93.78	90.86	90.54	90.00	85.54
4	Unknown composition AUROC standard deviation across sets (%)	0.35	0.73	0.44	1.04	0.65
5	Unknown composition AUROC min–max range (%)	93.2–94.1	89.8–91.6	89.9–91.0	88.4–91.2	84.5–86.1
6	Calibration ECE uncalibrated → calibrated (%)	6.67 → 3.06	NA	NA	NA	NA
7	Window-length tuning knee point	32	NA	NA	NA	NA
8	At 32 frames open-set metrics AUROC AUPR OSCR (%)	92.0 75.0 84.0	NA	NA	NA	NA
9	Latency at 32 frames stride 8 (ms per window)	15.0	NA	NA	NA	NA
10	AUROC retention under view shift (%)	96.8	95.1	94.5	93.4	90.1

Table 7 contains the statistical results corresponding to the key improvements presented in Section 6.1 and Section 6.2. In terms of accuracy, Macro-F1, AUROC and AUPR, the performance of the proposed model is improved consistently relative to the baselines. The stability of the obtained results is supported by bootstrap confidence intervals, while the statistical significance of the improvement is confirmed by the Wilcoxon signed-rank test (p < 0.05).

**TABLE 7:** Statistical summary of reported performance improvements.

Metric	Baseline	Δ improvement	95% bootstrap CI	Wilcoxon p-value
Accuracy, cross-subject	TCN baseline	+1.60 percentage points	+1.28 to +1.92	N/A
Macro-F1, cross-subject	TCN baseline	+3.20 percentage points	+2.92 to +3.48	N/A
Mean AUROC across five held-out sets	Softmax thresholding	+8.24 percentage points	+7.36 to +9.12	0.0313
AUPR, K = 10 held-out classes	Energy score baseline	+3.20 percentage points	+2.45 to +3.95	N/A

## 7 Conclusion

As demonstrated by the open-set hybrid CNN temporal network, collaborative robot perception can benefit from considering action understanding and novelty processing as a joint decision problem rather than an open-set classification problem with a *post hoc* threshold application. With a spatial encoder, efficient temporal aggregation, and an explicitly calibrated rejection gate using open-set scores, the system provides high recognition of known actions, along with the elimination of potentially unsafe false classifications of novel actions as one of known classes. Evaluation of the method on NTU RGB + D 120 with cross-subject and cross-view protocols and several configurations with unknown samples proves the effectiveness of the scoring and calibration process with a robust distinction of known and unknown actions and robustness to the unknown distribution shift. Future research will expand the framework along three directions that will be useful for practical applications to shared workspaces. First, we will evaluate the rejection scheme in the setting of robot-human interactions in the loop, by integrating the open-set gate with an existing safety supervisor and measuring the impact of different fallback policies on the time and cost of task completion under safety constraints. Second, we will tackle the issue of long-term drift in deployment by designing online adaptation strategies that allow learning of new thresholds and prototypes continuously without catastrophic forgetting. This way, the system will stay stable under the changes in operators, their tools, and work procedures. Third, we will increase the robustness of the solution to real shopfloor artifacts by adding multimodal streams to the training set, including depth or skeleton data, and employing domain-aware data augmentation for occlusions, motion blurring, and viewpoints changes present in real camera deployments in industrial settings.

## Data Availability

The data analyzed in this study are from the NTU RGB+D 120 Action Recognition Dataset provided by the ROSE Lab, Nanyang Technological University, Singapore, and are available to researchers subject to registration and the dataset release agreement. No new primary dataset was generated in this study. Further inquiries can be directed to the corresponding author.
